# Child and adolescent injury burden in the eastern mediterranean region: Findings from the Global Burden of Disease 1990-2017

**DOI:** 10.1186/s12889-020-08523-w

**Published:** 2020-04-03

**Authors:** Samar Al-Hajj, Charbel El Bcheraoui, Farah Daoud, Ibrahim Khalil, Maziar Moradi-Lakeh, Laith J. Abu-Raddad, Randa R. Hamadeh, Ali Mokdad

**Affiliations:** 1 Faculty of Health Sciences, American University of Beirut, Van Dyck Hall, PO Box 11-0236, Beirut, Riad El-Solh 1107 2020 Lebanon; 2 Institute for Health Metrics and Evaluation, University of Washington, Seattle, WA USA; 3 Weill Cornell Medicine-Qatar, Cornell University, Qatar Foundation, Education City, Doha Qatar; 4 Arabian Gulf University, Manama, Kingdom of Bahrain

**Keywords:** Child and adolescent health, Injury prevention, Eastern Mediterranean region, Global burden of disease

## Abstract

**Background:**

Child and adolescent injury is one of the leading causes of child death globally with a large proportion occurring in Low- and Middle-Income Countries (LMICs). Similarly, the Eastern Mediterranean Region (EMR) countries borne a heavy burden that largely impact child and adolescent safety and health in the region. We aim to assess child and adolescent injury morbidity and mortality and estimate its burden in the Eastern Mediterranean Region based on findings from the Global Burden of Disease (GBD), Injuries and Risk Factors study 2017.

**Methods:**

Data from the Global Burden of Disease GBD 2017 were used to estimate injury mortality for children aged 0–19, Years of Life Lost (YLLs), Years lived with Disability (YLDs) and Disability Adjusted Life Years (DALYs) by age and sex from 1990 to 2017.

**Results:**

In 2017, an estimated 133,117 (95% UI 122,587-143,361) children died in EMR compared to 707,755 (95% UI 674401.6–738,166.6) globally. The highest rate of injury deaths was reported in Syria at 183.7 (95% UI 181.8–185.7) per 100,000 population. The leading cause of injury deaths was self-harm and interpersonal violence followed by transport injury. The primary cause of injury DALYs in EMR in 2017 was self-harm and interpersonal violence with a rate of 1272.95 (95% UI 1228.9 – 1319.2) almost 3-times the global rate.

**Conclusion:**

Almost 19% of global child injury related deaths occur in the EMR. Concerted efforts should be integrated to inform policies and adopt injury preventive strategies to reduce injury burden and promote child and adolescent health and well-being in EMR countries.

## Background

Injury represents one of the leading causes of death and disability in the world, claiming more than 4·4 million lives in 2017 and imposing a substantial burden on global health [1]. Child and adolescent injuries preoccupy a sizeable portion of the injury landscape worldwide. As defined by the United Nations, “child” refers to 0–9 years and “adolescent” refers to 10–19 years of age [2]. Children and adolescents account for almost 30% of the world population. They are particularly vulnerable to injuries due their active and discovery driven nature in early years, and risk-taking behavior in adolescent years. Thousands of children saved from infectious diseases and malnutrition are killed or disabled as a result of injuries. According to the Global Burden of Disease 2017 study, an estimated 1900 children and adolescents die every day as a result of an injury [1].

The injury burden is disproportionally distributed globally with the majority of injuries occurring in low- and middle-income (LMIC) countries due to the lack of safety measures and appropriate health care facilities [3–6]. The Eastern Mediterranean Region (EMR) reports one of the highest rates of injury related deaths and disabilities among LMICs. According to the World health organization (WHO), the EMR encompasses 22 countries including Afghanistan, Bahrain, Djibouti, Egypt, Iran, Iraq, Jordan, Kuwait, Lebanon, Libya, Morocco, Oman, Pakistan, Palestine, Qatar, Saudi Arabia, Somalia, Sudan, Syria, Tunisia, United Arab Emirates, and Yemen. EMR countries sustain high rates of mortality and disability-adjusted life years (DALYs) particularly dues to injuries caused by road traffic crashes and violence [7–9]. Moreover, regional wars and political conflicts exacerbated the injury burden in the Middle Eastern Region, where millions of people are wounded by explosions while others displaced internally or forced to resettle as refugees in neighboring countries or elsewhere. Since the beginning of the ongoing wars, the region witnessed the world’s largest displacement crisis, with the vast majority of refugees and displaced individuals being children and adolescents below the age of 20 [10]. These refugees are particularly vulnerable to all types of injuries due to their diminished living conditions. Thousands of refugees, settling in overcrowded camps and harsh living situations, resorted to harmful coping mechanisms to survive including child labor and early marriages, thereby increasing their exposure to exploitation, violence and abuse [11–13].

This paper aims to assess the burden of children and adolescents’ injury in the EMR based upon findings from the Global Burden of Disease (GBD) 1990 to 2017 study. The article illustrates the magnitude and impact of injuries on children and adolescents in EMR countries and further presents evidence-based recommendations to inform policies and programs aiming at enhancing children and adolescents’ safety and well-being in this region.

## Methods

We adopted the Global Burden of Disease (GBD) methodologies and definitions of injury. Within the context of this study, injury refers to the physical harm and the psychological harm that hinder the individual’s potential well-being and growth [4]. The Global Burden of Disease classifies injuries under three main categories: 1. Transport Injury 2. Unintentional Injury, and 3. Self-harm and Interpersonal Violence. Injury morbidity and mortality were assessed using data from the Global Burden of Disease study 2017, with estimates of the world’s health for 359 diseases and injuries and 84 risk factors from 1990 to 2017 for 195 countries and territories and subnational locations for 16 countries. Based on the GBD methodology, Injury mortality data was estimated using multiple data sources including vital registration systems, verbal autopsies, registration systems, surveys, censuses, and demographic surveillance sites as well as police records and crime reports. Injury diagnoses and reported causes of injury associated deaths and disabilities were classified based on the International Classification of Disease (ICD), Ninth Edition (ICD-9) E Codes-Supplemental Classification of External Causes of Injury and Poisoning (E000- E999) and ICD tenth Edition ICD-10 codes V, W, S, Y and T. Injury classifications and their matching ICD 9 and ICD-10 codes have been published elsewhere [1].

Deaths, Years of Life Lost (YLLs), Years lived with Disability (YLDs) and Disability Adjusted Life Years (DALYs) by age and sex from 1990 to 2017 were assessed based on injury data accessed from GBD 2017. We used data from the Global Burden of Disease (GBD 2017), extracted from (https://vizhub.healthdata.org/gbd-compare/) and filtered by GBD injury categories for children aged 0–19 to allow us to compare between EMR countries, children ages and sex, as well as to enable us to observe injury trends over time. Tableau version 10.4 was used to visualize the GBD 2017 data for this study. Based on the GBD methodology, injury mortality data is reported as the age adjusted rate of injury associated death cause per 100,000 population [1]. Years of life lost (YLLs) is the measurement of years of life lost due to premature injury mortality. YLL refers to the sum of mortality data pertaining to each injury cause of death multiplied by standard expected individual life span at each age and Years lived with disability (YLDs) refers to years of life lived with any short or long-term health loss, and estimates the duration and severity of non-fatal injuries. Disability-adjusted life-years DALYs, defined as years of healthy life lost, is calculated based on estimation of data for fatal and non-fatal injuries. DALYs is the sum of years lost due to premature injury death (YLLs) and the years lived with disability (YLDs). Details of the YLLs and DALYs calculation and methodologies are published elsewhere [14–17]. Cause of injury death, by year, age and sex, were estimated using the Code of Death Ensemble model (CODEm) software.

We adopted the Socio-Demographic Index (SDI) to gain insights into the rates of injuries within the Eastern Mediterranean Region based on countries SDI. The SDI is a value between 0 and 1.0 calculated from the geometric mean of three rescaled components: total fertility rate under 25 (TFRU25), lag-distributed income per capita (LDI), and average educational attainment in the population over 15 years of age [1]. A least squares spline regression of mortality rate on SDI was used to represent the average relationship for the age group below 20. Additional SDI information are published elsewhere [1].

### Uncertainty analysis

We quantified and propagated uncertainty into final estimates by calculating Uncertainty Intervals (UIs) for cause-specific estimation components based on 1000 draws from the posterior distribution by age, sex, location, and year. 95% Uncertainty Intervals (UIs) were calculated using the 2·5th and 97·5th percentiles, and point estimates were calculated from the mean of the draws. Details on the estimation results and modeling processes are published elsewhere [1].

## Results

### Mortality

**Fig. 1:**
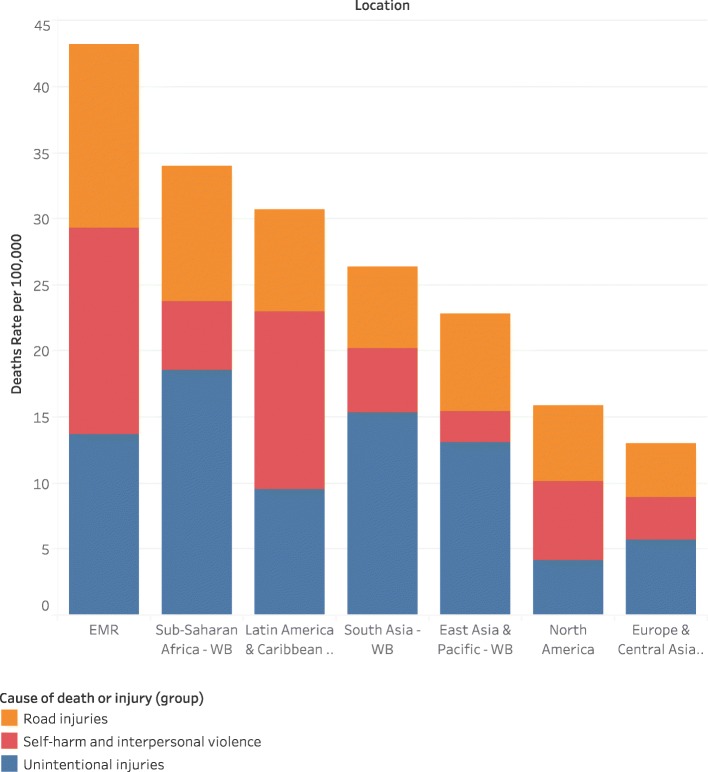
Shows the injury death rate for children 0–19 in EMR countries compared to the WHO regions

In the Eastern Mediterranean Region, an estimated 133,117 (UI 122,587-143,361) children died in 2017 compared to the global number of 707,755 (UI 674401.6–738,166.6). The rate of child and adolescent deaths in EMR was estimated at 43.2 (95% UI 40.8–45.6) per 100,000 population in 2017, well above the global rate of 27.2 (UI 25.9–28.4) (Fig. 1). Males experience a considerably higher mortality rate 51.7 (UI 48.5–55.2) compared to females 34.2 (UI 31.8–36.4), with a male-to-female ratio of 1.5. The highest rate of child and adolescent injury deaths in EMR was reported in Syria at 183.7 (UI 181.8–185.7) followed by Iraq at 80.1 (UI 77.1–83.6) and Yemen at 77.4 (UI 69.9–88.4). The lowest injury mortality rate was recorded in Bahrain at 10.2 (UI 9.1–11.4), Kuwait at 10.4 (UI 9.4–11.5) and Lebanon at 13.1 (UI 11.4–15.4) (Table 1).

**Table 1:** Death rates (1990, 2010 and 2017), YLLs, YLDs and DALYs by country for children and adolescents (0–19) injuries in the Eastern Mediterranean Region in 2017

EMR Country	Deaths Rate per 100,000				YLL 2017 Rate (per 100,000)	YLD 2017 Rate (per 100,000)	DALY 2017 Rate (per 100,000)	YLL/DALY 2017 ratio	YLL/YLD ratio	
	1990	2010	2017	% Change (1990–2017)					1990	2017
Afghanistan	113.6 (45.8–159.3)	80.4 (69.4–91.1)	50.6 (42.8–59.3)	−55.4	4028.8 (3390.8–4726.4)	436.6 (305.9–597.8)	4465.4 (3805–5166.5)	0.9	4.79	9.23
Bahrain	23.4 (20.9–25.8)	11.2 (10.3–12.1)	10.2 (9.1–11.4)	−56.5	795.4 (705.6–891.9)	186 (133.1–249.1)	981.4 (871.8–1100.2)	0.81	9.06	4.28
Djibouti	53 (33.8–65.2)	30.4 (23.1–39.9)	22 (16.6–32.1)	−58.5	1751.6 (1311.1–2571.6)	224.8 (165.6–293.7)	1976.4 (1530.3–2789.2)	0.89	14.91	7.79
Egypt	65.3 (48.5–78.2)	29.2 (25.7–33.5)	24 (20.4–28.2)	−63.3	1859.9 (1576.2–2207.1)	172 (125–228.7)	2031.9 (1740.7–2397.3)	0.92	23.29	10.82
Iran	137.6 (131.6–143)	50.5 (49.3–52.1)	30 (28.9–32.8)	−78.2	2316 (2229.9–2548.6)	173.7 (125.7–232.5)	2489.7 (2385–2721.7)	0.93	22.22	13.34
Iraq	57.1 (42.3–64.6)	48.7 (44.2–52.7)	80.1 (77.1–83.6)	40.4	6289.9 (6036.5–6577.6)	405 (285.4–557.2)	6694.9 (6410.5–7014.5)	0.94	4.65	15.53
Jordan	42 (36.1–46.5)	21.7 (19–24.6)	17.8 (14.7–21.3)	−57.6	1411 (1158.8–1697.6)	162.3 (116.9–217.7)	1573.3 (1316.3–1863.2)	0.9	15.76	8.69
Kuwait	73.7 (71.3–75.8)	14.6 (13.7–15.6)	10.4 (9.4–11.5)	− 85.9	797.4 (715.2–887.9)	182.1 (129.6–244.9)	979.5 (883–1086)	0.81	23.32	4.38
Lebanon	52.8 (48.9–57.4)	12.6 (10.8–14.7)	13.1 (11.4–15.4)	− 75.1	993.2 (858.8–1177.8)	186.8 (133.2–252.2)	1180 (1034.5–1365.5)	0.84	7	5.32
Libya	36.2 (30.2–42.7)	27.3 (22.5–32.4)	40.4 (36.1–45.2)	11.7	3100.3 (2766.9–3477)	326.3 (230.7–459.2)	3426.7 (3079.9–3839.8)	0.9	12.82	9.5
Morocco	57.9 (47.9–68.7)	28.5 (23.5–36.5)	23.1 (18.8–29.8)	−60	1784.1 (1441.3–2329.4)	181.6 (132.6–241.5)	1965.7 (1615.3–2509.8)	0.91	19.86	9.83
Oman	64.9 (44.3–79)	28.5 (25.4–31.8)	22.6 (19.5–25.5)	−65.3	1772.9 (1526.3–2009.8)	163.2 (116.7–220.9)	1936.1 (1688.5–2183.4)	0.92	25.7	10.86
Pakistan	52.9 (38–65.2)	47.6 (39.5–54.9)	36 (28.9–43)	−31.9	2825.3 (2273.4–3394.3)	210.7 (156.7–275.4)	3036 (2466.1–3609.3)	0.93	26.38	13.41
Palestine	52.4 (44.8–58.7)	30.3 (28.3–32.1)	15.7 (13.2–18.5)	−70	1233.8 (1036.4–1459.4)	315.9 (224.1–435.8)	1549.7 (1339.5–1792.7)	0.8	9.24	3.91
Qatar	32.5 (28.3–36.6)	19.6 (17.1–22.2)	15.6 (13.1–18.2)	−52.1	1188.8 (1000.9–1392)	173 (123.6–234.9)	1361.8 (1163.2–1577.3)	0.87	12.66	6.87
Saudi Arabia	51.3 (40.4–64.1)	22.3 (19–25.8)	21.2 (18.4–25.2)	−58.7	1596.9 (1381.5–1903.6)	180.6 (130.5–242.3)	1777.5 (1560.2–2089.6)	0.9	19.47	8.84
Somalia	93 (49.4–131.6)	71 (55.9–87.9)	63.4 (52.3–78.1)	−31.8	5049 (4139.1–6215.8)	345.4 (249–455.1)	5394.4 (4490.2–6604)	0.94	18.71	14.62
Sudan	144.2 (83.3–196.1)	59.9 (46–74.6)	44.2 (34.7–57)	−69.3	3576.9 (2765.3–4638)	207.1 (154.9–268.8)	3784 (2966.8–4843.6)	0.95	41.74	17.27
Syria	30 (24.3–35.6)	13.2 (11.9–14.5)	183.7 (181.9–185.7)	512	14,063.4 (13,924.2–14,214.2)	861.7 (585.3–1287.4)	14,925.1 (14,598.9–15,349.2)	0.94	10.91	16.32
Tunisia	53.8 (45.2–62.3)	24.1 (20.5–27.8)	16.7 (13.5–19.8)	−69	1292.6 (1035.2–1540.6)	173.6 (123.9–231.9)	1466.2 (1190.7–1720.9)	0.88	18.8	7.45
United Arab Emirates	39.6 (32.8–46.6)	39.5 9 (32.8–45.5)	25.5 (20.6–30.8)	−35.5	1952.6 (1569.1–2381.5)	187.9 (135.5–254.9)	2140.5 (1746.6–2565.8)	0.91	13.4	10.39
Yemen	95.4 (40.3–135.1)	47.1 (38.1–56.9)	77.4 (69.9–88.5)	−18.8	6114.1 (5496.9–7027)	283.9 (205–372.9)	6398 (5773.9–7321.1)	0.96	27.77	21.54

The highest number of child and adolescent deaths resulted from self-harm and interpersonal violence 48,051 (UI 46,046-49,386), followed by transport injury 42,829 (UI 38,573-47,233) and unintentional injury 42,236 (UI 37,967-46,741). Males death rate decreased from 92·2 (UI 75.1–105.5) in 1990 to 51.7 (UI 48.497–55.27) in 2017. Similarly, females experienced a major reduction in their total rate of injury related deaths from 60.1 (UI 48.03–69.1) in 1990 to 34.2 (UI 31.8–36.4) in 2017.

**Fig. 2:**
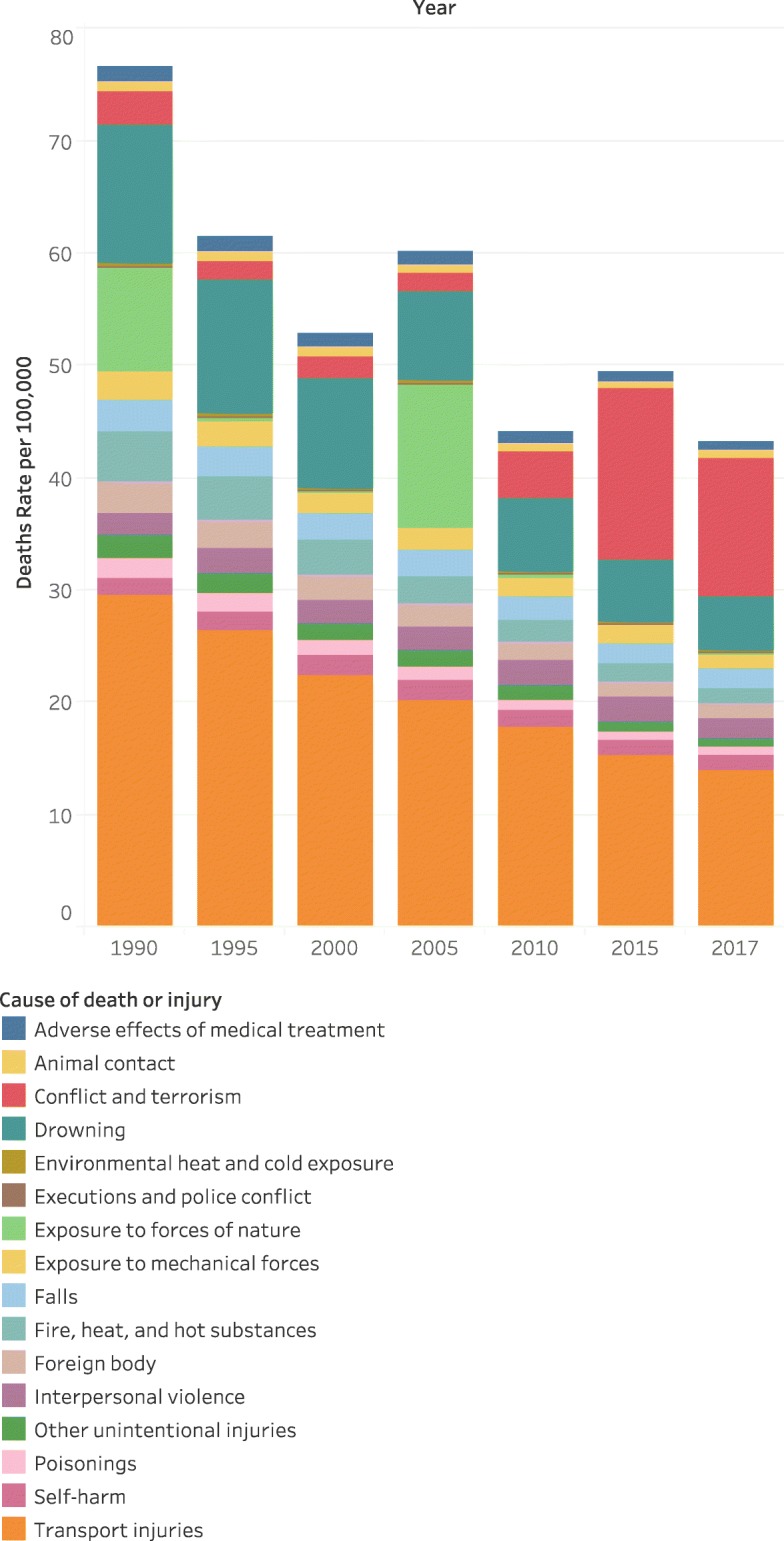
Depicts the change over time for injury mortality for children 0–19 in EMR from 1990 to 2017 (GBD 1990–2017)

**Fig. 3:**
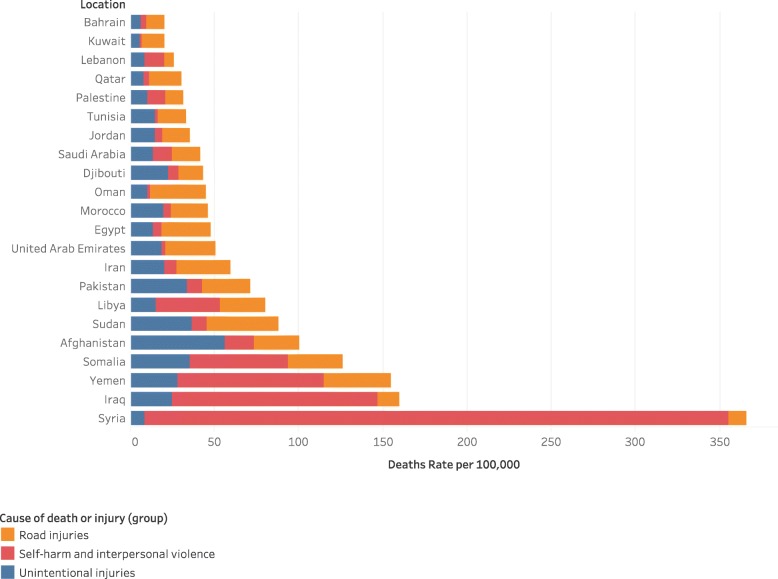
Displays the rate of death rate among different injury types for children 0–19 in EMR countries

Major variation in mortality rate was reported among EMR countries based on the type of injuries (Fig. 2). For transport injury, the highest rate of deaths was recounted in Sudan 21.4 (UI 15.4–30.04), for unintentional injuries, the highest rate was reported in Afghanistan 28.1 (UI 22.7–3.1) per 100,000 population and for self-harm and interpersonal violence, the highest death rate was recorded in Syria 173.9 (UI 173.5–174.4) per 100,000 population (Fig. 3).

### Years of life lost (YLLs)

In 2017, the rate of child and adolescent Years of Life Lost (YLLs) due to injuries was 3386.9 (UI 3196.6–3579.5) per 100,000 population in the EMR, exceeding the global rate of 2147.5 (UI 2042.8–2243.3) (Table 1). The leading cause of YLLs was self-harm and interpersonal violence reaching the rate of 1199.3 (UI 1150.4-1230.4) per 100,000 population.

Contrary to global patterns, the highest rate of YLLs was reported among adolescents aged [15–19] years and not [1–4] years, reaching the rate of 4427.9 (UI 4156.2-4706.4), approximately double the global rate of 2610.65 (UI 2511.4-2705.6).

The rate of YLL differs significantly across EMR countries. The highest rate of YLLs was recorded in Syria at 14,063.4 (13,924.2-14,214.2), followed by Iraq at 6289.9 (6036.5-6577.6), and Yemen at 6114.1 (5496.9-7027), while the lowest YLLs rates were reported in Bahrain at 795.4 (705.6–891.9), Kuwait at 797.4 (715.2–887.9) and Lebanon at 993.2 (858.8–1177.8), consecutively. It is worth noting that injury caused more YLLs than YLDs in EMR countries. The YLL was primary contributor to DALYs.

### Disability-adjusted life years (DALYs)

In 2017, injury caused 61,039,122.3 (UI 57,989,710.8-64,146,588.9) DALYs for children and adolescents in the EMR. Exceeding global rates, the Eastern Mediterranean Region estimated rate of DALYs was 3639.7 (UI 3425.8–3846.3) per 100,000 population in 2017, compared to the global rate of 2352.6 (UI 2235–2472.3). The leading cause of DALYs was self-harm and interpersonal violence with the excessive rate of 1272.95 (UI 1228.9 – 1319.2), almost 3-fold the global rate of 492.02 (UI 468.4–513.2) per 100,000 population), followed by unintentional injuries 1249.8 (UI 1121.7-1384.06) and transport injuries 1116.9 (UI 1011.8-1229.3).

Compared to 1990, child and adolescent DALYs witnessed a substantial reduction in rates between 1990 [6537.3 (UI 5350.6–7397.8)] and 2017 [3639.7 (UI 3425.8–3846.3)]. The rate of DALYs decreased in 2017 in some EMR countries including Sudan (12,232.5), Iran (11,437.1), and Afghanistan (11,186.1), while increased tremendously up to six folds its early rate in other EMR countries including Syria (14,925.1), Iraq (6694.9) and Yemen (6397.9).

### The socio-demographic index (SDI)

In 2017, in EMR countries Socio-Demographic Index (SDI) spans from 0.29 in Afghanistan to 0.79 in Kuwait and the United Arab Emirates. EMR countries with low SDI sustain a high toll of child injury related deaths and DALYs rates exacerbated by ongoing regional war and conflicts including Syria [183.6 (UI 181.8–185.6)], Iraq [(80.12 UI 77.1–83.6)] and Yemen [(77.4 (UI 69.9–88.4)). Contrary to global patterns, EMR countries with high SDIs suffer from excessively high rates of DALYs despite being at peace, mainly because of transport related injuries including United Arab Emirates [1169.2 (UI 918.3–1441.3)] and Oman [1341.2 (1096.8–1550.2)].

## Discussion

Our study shows a high burden of child and adolescent injury morbidity and mortality in the Eastern Mediterranean region. Accounting for almost 8% of the global population, The EMR population bears up to 19% of global child injury related deaths in the world. Injury represents the leading causes of child and adolescent deaths in EMR, surpassing deaths caused by major diseases combined including Malaria, tuberculosis and HIV [1]. In addition to loss of lives, injuries are major contributors to morbidity and lifelong disabilities. EMR countries recorded high rates of YLLs, YLDs and consequently DALYs, exceeding corresponding global rates. Consequently, sustaining these high rates will burden the overwhelmingly young generation in the Eastern Mediterranean region (40% of its 600 million individuals below the age of 20) [18], and will impose a devastating ripple effect on millions of children in the region for decades to come.

Findings from this study’s systematic analyses of child and adolescent injuries shows an evident decrease in the injury mortality rate in EMR countries across time from 1990 to 2017. Nevertheless, this declining rate varies according to mechanisms of injury. While an apparent reduction was reported in death rates for ‘transport injury’ and ‘other unintentional injuries’, ‘self-harm and violence’ injury showed a significant upsurge in mortality rate, more than a 3-fold increase over time from 1990 to 2017. In the first 2 decades 1990–2010, all EMR countries, with no exception, witnessed a clear reduction in injury morbidity and mortality rates. Nevertheless, with the political unrest and protracted conflicts, many countries showed a turning point during the 2010–2017 period of time with sharp resurged inclines in injury morbidity and mortality rates, particularly among regions suffering from wars and conflicts including Syria, Iraq and Yemen that recounted a considerable increase in their death rates from 2010 to 2017, reaching in some countries up to 14-fold its 2010 deaths status.

Despite its human and economic impact, injury remains a neglected public health problem in the EMR due to multiple reasons, including mainly protracted regional wars, perpetual political instabilities and conflicts that continue to strongly distract regional health policies and priority programs. Although the rates of injuries in EMR have decreased over the last two decades, the number of child mortality and morbidity associated with war and violence had increased at an unprecedented rate over the last few years, overpassing global regions suffering similar instabilities and violence including African countries. Despite the substantially high number of children killed in African region as a result of internal conflict and sectarian violence [19], the rates of child deaths in EMR countries remain excessively high in EMR countries like Iraq, Yemen and Syria where the reported child deaths rate were 2.8, 4 and 11 times respectively higher than the rates in African countries [1].

Road traffic injuries represent another major threat to the health and well-being of children and adolescents, particularly in high income EMR countries. Compared to global estimates, EMR reported high rates of road crashes deaths and injuries mainly due various factors that exacerbate current road hazards and render the environment adversely unsafe and risky and lead to a substantial increase in the number of road crashes responsible for fatalities and long-term disabilities, particularly among the adolescent population [7–9, 20–25].

Our study revealed that EMR countries with different SDI levels have large disparities in injury death rates. In the EMR, even countries with high SDI have higher burden from injuries compared to countries with the same level of development. These patterns maybe due to higher risk factors, poor access to health care, and lack of quality health care in these countries. Indeed, these findings call for increased programs and interventions to address the burden of injuries in the EMR region among all countries. Moreover, countries with high SDI and financial means are urged to better address the burden and to have more focused interventions from prevention, response, and treatment.

Concerted efforts and multi sector approaches should be integrated to save lives and control injuries in EMR. In this paper we propose six recommended strategies that can be adopted to effectively reduce injuries and mitigate their impacts on the child and adolescent population:

1. **War and Conflict Emergency Plan:** The high rates of children and adolescents being victims of self-harm and violence in EMR reflect the destructive impact of armed conflicts on children and adolescents in the region. It is imperative to provide children special protection during wars and man-made disasters to prevent premature deaths and serious injuries. Emergency preparedness plans and child protection humanitarian response should be activated at the onset of any crises (i.e. war, armed conflicts) [26, 27]. Adequate war evacuation plan should be communicated along with well-trained personnel, government support and public awareness to provide safe shelters and to avoid children exposure to violence and abuse. An urgent call for international leaders, organizations and human rights agencies to mobilize their efforts and end war and violence and their repercussions on children (e.g. child soldiers, child abuse and sexual exploitation) and safeguard children and youth whose lives and well-being are largely threatened.
2. **Policies, Laws and Enforcement:** Informing injury prevention policies and laws are pivotal to reducing injuries. Enforcement of safety regulations is key to curtail injury-associated deaths and reduce serious injuries. Many EMR countries have safety laws and legislations in place, however lack of enforcement adversely limits their effects on reducing injury morbidity and mortality. There is an urgent need for EMR to implement and enforce safety policies to control for many types injuries, including transport related injuries- a largely preventable and leading cause of deaths among adolescents.
3. **Emergency Medical Services and Healthcare Access:** The absence of responsive and timely Emergency Medical Services (EMS) negatively affect patient survival and outcome [28]. EMR countries should adopt strategies to enhance their emergency medical services and improve injury response time. EMR Countries with low SDI and high injury rates would benefit from an enhanced Emergency service to care for injured children and reduce preventable disabilities. Access to quality healthcare services is another major factor that contributes to enhancing injury survival rates. There is an urgent need for a systematic effort to address multiple social and environmental barriers (funding, rural service provision) and improve access to healthcare services in EMR countries. EMR countries should ensure individuals entitlement to universal health coverage to ensure health service accessibility from injury prevention, to treatment to rehabilitation [29, 30].
4. **Injury Surveillance Systems:** A limited number of EMR countries have national injury surveillance systems and trauma registries [31–34]. Existing data are hospital administrative, clinical records and police reports. Readily accessible data lacks accurate injury coding and documentation of many essential information critically relevant to understand the magnitude, setting and context of injury and its risk factors and consequently limit injury research impact on prevention and policies. There is a driving need for robust and efficient injury surveillance systems and coding to resourcefully capture comprehensive injury data in EMR countries. Building capacity among health professionals and seeking buy-ins from key stakeholders are vital for the successful integration of injury surveillance systems at local hospitals. Moreover, these surveillance systems will constitute the backbone for data driven injury research in EMR countries [35, 36]. Injury research is necessary to accurately understand trends and patterns of injury in the region, to constructively generate evidence-based knowledge and to provide insights into the development of injury interventions strategies and educational awareness programs.
5. **Education and Awareness:** Educational and awareness programs play a pivotal role in improving people’s knowledge and informing them about various mechanisms of injuries and their associated risk factors [37]. Awareness programs can motivate behavior change within communities and promote children’s safety. Strengthening communication, dialogue and partnership with local ministries and NGOs are essential to initiate and implement awareness and educational programs to promote injury free environment for children and adolescents. Awareness programs should integrate evidence-based injury interventions and strategies at multiple levels to make significant progress towards improving child and adolescent health and safety in the EMR.

There are limitations to this study. Firstly, we are using GBD injury categorization and are limited to these categories for our study analyses. Nevertheless, since GBD adopts a systematic approach, these categories allows us to compare between countries of the Eastern Mediterranean Region, children ages, sex, and display trends of injuries over time. Secondly, the study analysis mainly relies on available yet limited EMR injury data. Many EMR countries lack injury surveillance programs, vital statistics, health information systems and trauma registries. Existing hospital administrative and police reports data are inherently limited and suffer from major shortcomings when reporting injury morbidity and mortality. War-affected EMR countries are far more prone to the absence of reliable and well-documented health data. Consequently, many modeling techniques were adopted to fill in this gap in data and estimate the injury burden in EMR countries. Thirdly, the study findings are solely reporting the physical damage sustained by injuries without accounting for the indirect effects of injuries including the psychosocial impact of injuries on the health and well-being of children and adolescents in EMR and its repercussions on their mental health. Fourthly, the number of sustained interpersonal violence may be inaccurate, as it has the tendency of being potentially underreported due to the nature of violence in some EMR countries under fire and the possibility of cover up in other countries with restricted reporting of sensitive information.

This study complements exhibiting studies addressing the injury health problem in the Eastern Mediterranean Region [7, 8, 21]. It highlights the alarming number of child fatalities as a result of transportation injuries, unintentional injuries and self-harm and violence. Recommendations of this study will transfer knowledge to key stakeholders including health professionals, policy makers, and law enforcement personnel to define specific points of countermeasures interventions, to develop health awareness programs and to deliver evidence-based injury preventive policies and programs, ultimately contributing to the reduction of injuries and the improvement of child and adolescent health and wellbeing in EMR.

## Conclusions

This study analyzed and documented the burden of children and adolescents’ injury morbidity and mortality in the Eastern Mediterranean Region. EMR bares one of the highest rates of child and adolescent injuries and deaths globally. These injuries impose tremendous strains on local health care systems, depleting their infrastructure and limited resources. The noticeable massive number of child morbidity and mortality was impacted by few war-ridden countries and presented a critical indicator on children and adolescents threatened safety and well-being in the region. Reducing child and adolescent injury morbidity and mortality is of utmost importance to substantially lessen the burden on healthcare systems and to secure prosperity and thriving future for this region. Child and adolescent threatened health adversely affects the region’s social and economic progress, particularly with deaths and disabilities affecting the economically active and productive youth population. Findings from this study urge for immediate actions to be taken to protect children in armed conflicts and war zones and to develop and implement safety policies and strategic preventive measures to reduce child injuries and mitigate their impacts on EMR countries.

## Data Availability

The datasets generated and/or analysed during the current study are available in the GBD Compare repository, https://vizhub.healthdata.org/gbd-compare/

## Abbreviations

GBD: Global Burden of Disease
LMIC: Low- and middle-income
WHO: World health organization
EMR: Eastern Mediterranean Region
ICD: International Classification of Disease
YLL: Years of Life Lost
YLD: Years lived with Disability
DALYs: Disability Adjusted Life Years
CODEm: Code of Death Ensemble model
SDI: Socio-Demographic Index
UI: Uncertainty Interval
EMS: Emergency Medical Services
NGO: Non Governmental Organization
